# Variation and asymmetry in host-symbiont dependence in a microbial symbiosis

**DOI:** 10.1186/s12862-018-1227-9

**Published:** 2018-07-09

**Authors:** Ewan J. A. Minter, Chris D. Lowe, Megan E. S. Sørensen, A. Jamie Wood, Duncan D. Cameron, Michael A. Brockhurst

**Affiliations:** 1 Department of Animal and Plant Sciences, University of Sheffield, Alfred Denny Building, Western Bank, Sheffield, S10 2TN UK; 2 Centre for Ecology and Conservation, University of Exeter, Penryn, TR10 9FE UK; 3 Department of Biology, University of York, York, YO10 5DD UK; 4 Department of Mathematics, University of York, York, YO10 5DD UK

## Abstract

**Background:**

Symbiosis is a major source of evolutionary innovation and, by allowing species to exploit new ecological niches, underpins the functioning of ecosystems. The transition from free-living to obligate symbiosis requires the alignment of the partners’ fitness interests and the evolution of mutual dependence. While symbiotic taxa are known to vary widely in the extent of host-symbiont dependence, rather less is known about variation within symbiotic associations.

**Results:**

Using experiments with the microbial symbiosis between the protist *Paramecium bursaria* and the alga *Chlorella*, we show variation between pairings in host-symbiont dependence, encompassing facultative associations, mutual dependence and host dependence upon the symbiont. Facultative associations, that is where both the host and the symbiont were capable of free-living growth, displayed higher symbiotic growth rates and higher per host symbiont loads than those with greater degrees of dependence.

**Conclusions:**

These data show that the *Paramecium-Chlorella* interaction exists at the boundary between facultative and obligate symbiosis, and further suggest that the host is more likely to evolve dependence than the algal symbiont.

**Electronic supplementary material:**

The online version of this article (10.1186/s12862-018-1227-9) contains supplementary material, which is available to authorized users.

## Background

Symbiosis —the intimate living together of unlike organisms— is a major source of evolutionary innovation, providing interacting species with new functions and thus facilitating the evolution of complex life [1, 2]. Symbioses are common in nature, and, by allowing species to exploit otherwise inaccessible ecological niches, underpin the diversity and functioning of natural ecosystems [3–5]. Yet understanding the origins and evolutionary stability of symbioses remains a major challenge for evolutionary biologists. The evolutionary transition from free-living to obligate symbiosis requires that the fitness interests of interacting species be aligned, and that the species evolve to become mutually dependent [6–10]. However, while famous examples of obligate symbiotic partnerships exist, many symbioses are facultative wherein species retain the ability to survive in the free-living state [11, 12]. Comparative evolutionary analysis suggests that this variation among lineages in their degree of host-symbiont dependence is at least partially explained by the types of benefits exchanged between symbiotic partners and the mode of symbiont inheritance, with mutual dependence being more common in vertically-inherited, nutritional symbioses [13]. These macroevolutionary patterns cannot reveal, however, the extent of variation in host-symbiont dependence available to natural selection within symbioses, nor the potential for asymmetries in dependence among partners in a symbiosis.

Photosynthetic endosymbioses (photosymbioses), typically between eukaryotic algae and animal or protist hosts, are a classic example of widespread and ecologically important symbiosis [5, 12, 14] and therefore represent a useful model system for understanding evolutionary transitions in symbiosis. Photosymbioses are typically based upon the reciprocal exchange of nutrients in the form of fixed carbon from symbiont to host, and nitrogen compounds from host to symbiont [15]. Photosymbioses vary widely in their degree of host-symbiont dependence, from ancient and obligate organelles (e.g. primary, secondary, and tertiary plastids in eukaryotic algae, see Keeling [16]), to facultative symbioses where symbiotic partners are also able to survive in the free-living state (e.g. *Symbiodinium* and anthozoan corals [17, 18]). Across the extant eukaryotic tree of life, transitions from facultative to obligate photosymbiosis have occurred independently a number of times [16, 19, 20], yet facultative photosymbioses are arguably both more common and more diverse [12, 21]. Little is known, however, about variation in host-symbiont dependence within facultative photosymbioses and the ecological drivers selecting for maintenance of the facultative habit.

The microbial photosymbiosis between the host *Paramecium bursaria* —a heterotrophic ciliate— and the symbiont *Chlorella* sp. —a green alga— is a tractable model system [22–27] where the fitness effects of symbiosis relative to free-living can be directly quantified [28]. The *P. bursaria-Chlorella* (Pb-C) symbiosis is widespread in shallow freshwater habitats, and is primarily based upon provision of nitrogen compounds from host heterotrophy to the symbiont, and of maltose and oxygen derived from symbiont photosynthesis to the host [29–32]. The Pb-C symbiosis has evolved multiple times, such that, whilst each Pb-C strain contains a clonal population of *Chlorella*, multiple origins of symbiotic lineages occur across the *Chlorella* clade [33, 34]. Within the *Chlorella* clade, *C. vulgaris* are found in both the free-living and symbiotic states, whereas *C. variabilis* is more typically associated with symbiosis [35]. We have previously shown for a single Pb-C pairing that the fitness effects of symbiosis are environmentally context dependent and highly asymmetric: For hosts, symbiosis is costly in the dark but becomes increasingly beneficial with increasing irradiance, whereas, for symbionts, symbiosis is not beneficial and becomes increasingly costly with increasing irradiance [28]. Hosts exert tight control over symbiont load (i.e., the number of symbionts per host cell), regulating symbiont number in relation to light to maximise the benefit-to-cost ratio of symbiosis [28, 36] . Accordingly, symbiont load peaks at low light levels but is reduced both in the dark, where symbionts are not beneficial, and at high light levels, where per symbiont benefits are highest [28]. Given the inherent conflict between these symbiotic partners, and the strong environmental context dependence of the fitness effects of symbiosis, we hypothesise that selection to retain free-living growth should be stronger for *Chlorella* than *P. bursaria* due to the asymmetries in the fitness benefits of symbiosis.

Here we experimentally investigate natural variation in host-symbiont dependence by comparing free-living versus symbiotic growth among five Pb-C pairings. We report variation in both the fitness effects of symbiosis and host-symbiont dependence between Pb-C pairings. Among the five Pb-C pairings, we observed fully facultative associations, an association displaying mutual dependence, and associations in which hosts alone displayed dependence. Notably, symbiotic growth rates were higher in Pb-C pairings that retained the fully facultative lifestyle and maintained higher symbiont loads. Our data therefore show that Pb-C pairings vary in the degree of host-symbiont dependence, and suggest that *Paramecium* is more likely to evolve dependence than *Chlorella*.

## Materials & methods

### Paramecium strains and culturing conditions

Experiments were performed using five *Paramecium bursaria* strains along with their naturally occurring *Chlorella* symbionts. These Pb-C pairings are designated 186b, HA1, HK1, CT39, and Dd1. 186b (CCAP 1660/18) was obtained from the Culture Collection for Algae and Protozoa (Oban, Scotland) and isolated in the UK, whilst the remaining four pairings were all obtained from the Paramecium National Bio-Resource Project (Yamaguchi, Japan) and were all isolated in Japan. Further details of the Pb-C pairings used are provided in Additional file 1: Table S1. All experiments were performed by culturing in bacterized Protozoan Pellet Media (PPM, Carolina Biological Supply, NC, USA) which was made to a concentration of 0.66 g L^− 1^ with Volvic natural mineral water, and inoculated approximately 20 h prior to use with *Serratia marcescens* from frozen glycerol stocks. All stock cultures were maintained at 25 °C with 50 μE m^− 2^ s^− 1^ of light and a 14:10 L:D cycle. Stock cultures were maintained by batch culture, where cultures were diluted by half every 2–3 weeks with fresh bacterized PPM. Unless otherwise stated, experiments were performed under the same culture conditions.

### Symbiotic and apo-symbiotic host growth rates in response to light

Growth rates of hosts were compared across a light gradient and in the presence (symbiotic) or absence (apo-symbiotic) of *Chlorella* symbionts. Apo-symbiotic cell cultures were established by treating symbiotic cells with a combination of paraquat (10 μg mL^− 1^) and cyclohexamide (10 μg mL^− 1^) and exposing the cells to high light intensities (> 50 μE m^− 2^ s^− 1^) for a period of between four and seven days, until host cells were visibly symbiont free. Apo-symbiotic cell cultures were verified by monitoring the colour of host cells on the microscope, and observing that re-greening by *Chlorella* did not occur over three weeks.

Both symbiotic and apo-symbiotic *P. bursaria* cells were washed and concentrated using sterile Volvic and re-suspended in bacterized PPM. Cells were acclimated to 50 μE m^− 2^ s^− 1^ light for two days before being washed once again, re-suspended in fresh bacterized PPM. Cells were then acclimated to their treatment light condition (0, 1.5, 3, 6, 12, 25, & 50 μE m^− 2^ s^− 1^) for five days before being washed and re-suspended in bacterized PPM at a target cell density of approximately 350 cells mL^− 1^. To estimate growth rates, cell densities were determined at 0, 24, and 48 h by fixing 350 μL of each cell culture, in triplicate, in 1% *v*/v glutaraldehyde in 96-well flat bottomed micro-well plates. Images of each well after settling were recorded using a Nikon D600 camera mounted to an inverted microscope through a 4× objective lens. Cell counts for each well were recorded using an automated image analysis macro in ImageJ v1.50i [37].

### Free living Chlorella growth

Free-living algal cultures were established in triplicate by washing 10 mL of stock culture in approximately 200 mL of sterile Volvic on an 11 μm nylon mesh. Host cells retained by the mesh were re-suspended in 1.5 mL Volvic and ultra-sonicated using a Fisherbrand Q500 Sonicator (Fisher Scientific, NH, USA), at a power setting of 20% for 10 s. Ultra-sonication resulted in lysis of host cells (confirmed by visual inspection) and release of symbionts into the surrounding media. Algal symbionts were separated from host cell lysate by centrifugation, re-suspended in 5 mL Bold’s Basal Medium (BBM) [38], and cultured in 30 mL glass tubes under the same conditions as for host stock cultures but with the addition of shaking at 130 rpm. The dynamics of these populations were tracked for five days. Cell densities were estimated each day using a CytoFLEX S flow cytometer (Beckman Coulter Inc., CA, USA), and manually gating *Chlorella* events for each individual sample using CytExpert2.0 (Beckman Coulter Inc., CA, USA). Specifically, *Chlorella* cells were distinguished from other particles on the basis of their fluorescence and size characteristics, which were initially determined by visual inspection of a subset of the flow cytometry data.

### Host symbiont load in response to light

*P. bursaria* cells with symbionts were washed and concentrated using sterile Volvic and re-suspended in bacterized PPM. Cells were evenly split into 28 microcosms each containing 5 mL of bacterized PPM and microcosms were randomly assigned to one of seven light treatment groups (*n* = 4). Microcosms were acclimated to their light treatment (0, 1.5, 3, 6, 12, 25, & 50 μE m^− 2^ s^− 1^) for approximately 6 days prior to flow cytometry analysis.

Host symbiont loads were estimated using a CytoFLEX S flow cytometer (Beckman Coulter Inc., CA, USA) by measuring the intensity of chlorophyll fluorescence for individual *P. bursaria* cells (excitation 488 nm, emission 690/50 nm). Data are presented as relative fluorescence, and are calibrated against 8-peak beads, to reduce variation between samples run in separate sessions.

### Data analysis

All statistical analyses were performed in R v.2.3.4 (R Core Development Team, 2016). The raw data analysed here is provided in Additional file 2, and further details of the fitted statistical models are provided in Additional file 3. Host growth rates were analysed treating light as either a continuous variable or a factor (the results of both analyses were qualitatively similar). In the first analysis, strain, symbiont presence/absence, and light were treated as factors. In the second analysis, since the relationship between growth and light differed markedly for symbiotic and apo-symbiotic hosts, we analysed these responses separately to detect strain-specific differences in growth using linear and non-linear regression for apo-symbiotic and symbiotic responses, respectively. Symbiotic host growth responses were modelled as:

$$ r=\frac{r_{max}\left(L-{p}^{\prime}\right)}{k+\left(L-{p}^{\prime}\right)} $$

where *r* is growth rate at a given light intensity (*L*), *r*_*max*_ is the light dependent maximum growth rate, *k* is the half saturation constant and *p*^′^ is the threshold light concentration (i.e. light concentration when growth is zero). Free living symbiont growth rates were analysed by One Way ANOVA.

Host symbiont loads were analysed by non-linear regression and non-linear mixed effects models (NLME) with the function

$$ \phi =\frac{a\ \left(L-{l}^{\prime}\right)}{b+{\left(L-{l}^{\prime}\right)}^c} $$

where *ϕ* equals the mean host symbiont load (relative units of chlorophyll host^− 1^) at a given light intensity (*L*), *a*, *b*, *c*, and *l* are parameters.

## Results

**Fig. 1:**
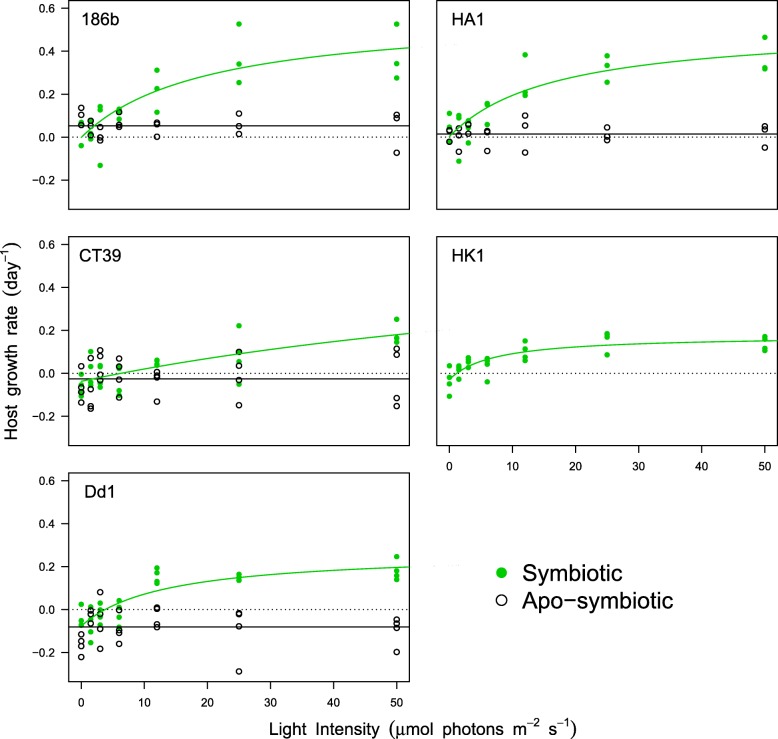
Reaction norms for host growth rate (day^− 1^) in response to light (μE m^− 2^ s^− 1^), for both symbiotic (green) and aposymbiotic (open) hosts, with fitted models (mean growth for aposymbiotic, non-linear regression for symbiotic). Each panel shows data for a different strain. Dotted line indicates where host growth equals zero

To examine variation in the effect of symbionts on host growth, we grew multiple independent strains of *P. bursaria* across a light gradient, both with and without symbionts. Growth rates for hosts with symbionts increased with light, whereas growth rates for hosts without symbionts were unaffected by light levels (light by symbiosis interaction, F_1,213_ = 69.3, *P* < 0.001), and the effect of symbionts on host growth varied between host strains (strain by symbiosis interaction, F_3,213_ = 3.5, *P* = 0.009). To further understand these patterns, growth responses of hosts with and without symbionts were analysed separately (Additional file 3). For all host strains, symbiotic host growth rates were either zero or negative in the dark and increased as a function of light, in most host strains this response was asymptotic reaching a maximum growth rate at high light levels (Fig. 1). Host strains HA1 and 186b with symbionts had the highest maximum growth rates (*r*_*max*_) and were significantly higher than in host strain HK1 with symbionts (two-sample t-tests: HK1 vs HA1, *t* = 3.104, *P* = 0.039; HK1 vs 186b, *t* = 3.097, *P* = 0.036). Host growth rates without symbionts varied between symbiont-free host strains (ANOVA, F_3,94_ = 15.0, *P* < 0.001), from low (HA1 & 186b) to negative growth rates (CT39 & Dd1), and did not respond to light (ANOVA, F_6,88_ = 0.57, *P* = 0.757). For one host strain, HK1, hosts without symbionts did not survive. These data suggest that host strains varied both in the benefit derived from symbiosis and in their dependence upon symbionts for growth and survival.

**Fig. 2:**
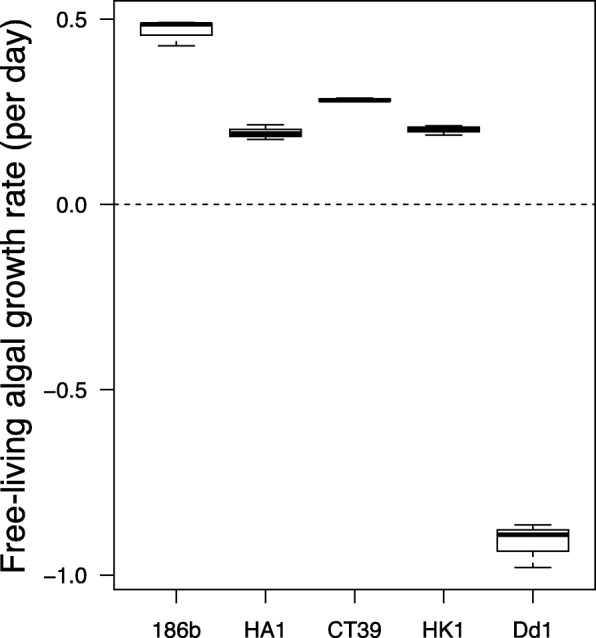
Growth rate of extracted *Chlorella* symbionts in 7-day cultures grown in Bold’s Basal Medium immediately following mechanical liberation from *Paramecium bursaria* hosts. Boxes show median and ranges for three independent culture replicates, dotted line indicates zero growth

To estimate survival of algal symbionts in the free-living state, *Chlorella* were isolated from their host and grown for one week in 50 μE m^− 2^ s^− 1^ light and population densities measured daily*.* Algal strains varied in their free-living growth rate (Fig. 2, ANOVA, F_5,12_ = 767, P < 0.001). Four algal strains displayed positive growth rates, whereas algae isolated from Dd1 were unable to grow in the free-living state (Fig. 2). Taken together with the data for free-living host growth rates, these data suggest that whereas some Pb-C pairings were facultative, wherein both the host and symbiont were capable of free-living (186b and HA1), other Pb-C pairings displayed some degree of dependence. For example, both the host and symbiont from the strain Dd1 were mutually dependent (i.e., unable to sustain free-living growth), while in CT39 and HK1 the symbionts were capable of free-living but the hosts were not, suggesting host dependency upon symbionts.

**Fig. 3:**
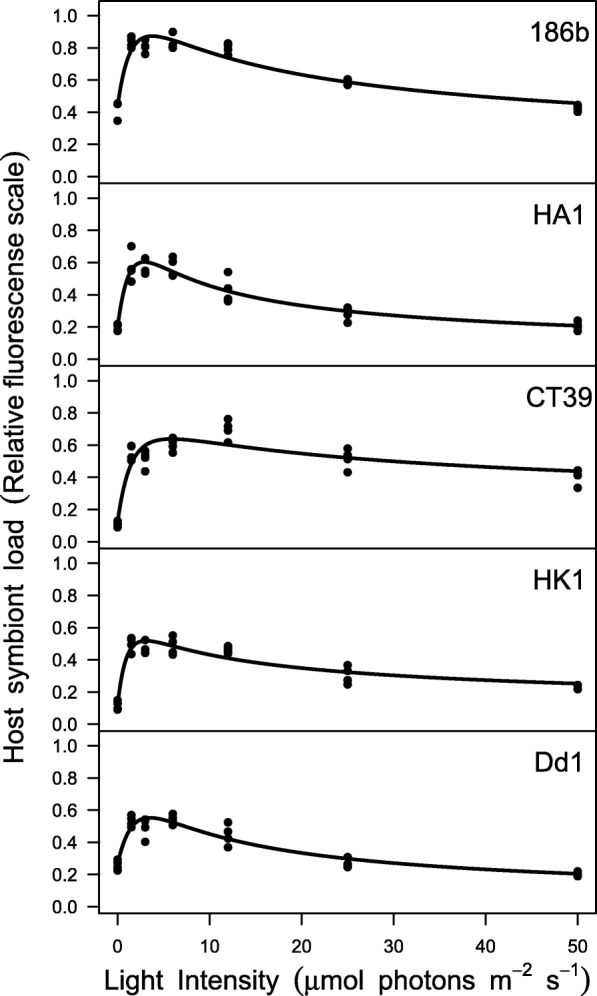
Reaction norms of mean host symbiont load (estimated from individual host chlorophyll fluorescence, scale is relative fluorescence) in response to light (μE m^− 2^ s^− 1^), for symbiotic hosts. Each panel shows data for a different strain

We previously showed that hosts tightly regulate symbiont load in relation light level to maximise the benefit-to-cost ratio of symbiosis [28]. To test whether host control varied among Pb-C pairings and was related to the degree of host-symbiont dependence we measured the per host symbiont load of each Pb-C pairing across a light gradient. Consistent with our previous finding, across all hosts, symbiont loads were lowest in the dark, peaked at low light intensities (2–8 μE m^− 2^ s^− 1^), and declined to intermediate levels at high light intensities (Fig. 3). While this pattern of symbiont load was broadly consistent among hosts, we did observe minor variations in the estimated parameters of the fitted curves (NLME, χ^2^_6_ = 118, *P* < 0.001; see Additional file 3). Specifically, host strain 186b had a higher symbiont load than HK1 independent of light level (i.e. parameter *a*). Peak symbiont load occurred at lower light intensities in host strains 186b and Dd1 than HK1 and CT39 (i.e. parameter *l*), potentially suggesting differences in the light environment to which the Pb-C pairings were adapted in nature. Host strains Dd1 and HA1 reduced symbiont load at high light intensities to a greater extent than host strain CT39 (i.e. parameter *c*), suggesting variation in the intensity of host regulation of symbiont load. These data suggest that host control is a broadly conserved trait across *P. bursaria*, but show no clear association between host control parameters and host-symbiont dependence, except that symbiont load was highest in the most facultative host (186b) and lowest in the host least able to survive without its symbionts (HK1).

## Discussion

The transition from facultative to obligate symbiosis, and thus the evolution of mutual dependence constitutes a major evolutionary transition in individuality [8, 9], and underpins the evolution of cellular organelles such as chloroplasts [1]. The evolutionary transition to mutual dependence requires there to be variation in host-symbiont dependence available for natural selection to act upon, and for mutual dependence to be associated with higher symbiotic fitness [6–10]. Using experiments with the microbial photosymbiosis between the ciliate host, *P. bursaria*, and the green alga, *Chlorella* sp., we demonstrate variation in host-symbiont dependence ranging from Pb-C pairings that are fully facultative to those that display either mutual dependence or dependence of hosts upon symbionts. Thus the *P. bursaria-Chlorella* interaction appears to exist on the boundary between facultative and obligate symbiosis. Moreover, since symbiotic growth rates of facultative Pb-C pairings were higher than those showing greater degrees of dependence—indeed the host HK1 which was unable to survive without symbionts showed the lowest symbiotic growth rate—it seems likely that facultative symbiosis may be favoured by selection. Interestingly, this is consistent with the distribution of symbiotic strains across the predominantly free-living *Chlorella* clade [34], which suggests repeated transitions from free-living to symbiosis and a long evolutionary history of its association with *P. bursaria* being facultative. Furthermore, Pb-C pairings that were more recently isolated from natural populations (186b and HA1 were isolated in 2006 and 2010, respectively; Additional file 1: Table S1), were more facultative than those with longer histories of laboratory culture (HK1 and Dd1 were isolated in 1990 and 1995, respectively; Additional file 1: Table S1). This could suggest that host-symbiont dependence is a derived trait among lab-adapted Pb-C pairings, whereas in natural populations the facultative state is more common, however more extensive studies of natural populations will be required to test this hypothesis.

Dependence was more commonly observed in *P. bursaria* than in *Chlorella*, possibly suggesting an asymmetry in selection for dependence between the host and the symbiont in this system. This would be consistent with our previous work, which showed that this is an exploitative symbiotic interaction, wherein hosts benefit more than symbionts from engaging in symbiosis [28]. This underlying conflict between host and symbiont would be expected to select for the retention of free-living ability, particularly in the symbiont. The fitness benefit of symbiosis to hosts increases with increasing light intensity and with decreasing availability of heterotrophic food [28], suggesting that selection for dependence in hosts is likely to be environmentally context dependent. We would predict therefore that *P. bursaria* should be more likely to evolve dependence on their symbionts in high light, low food habitats, but less likely in low light, high food habitats, or in environments that are highly variable in terms of light intensity and/or food availability. Indeed, in variable environments the facultative nature of the photosymbiosis may allow for partner-switching whereby hosts could acquire locally-adapted symbionts to promote their invasion of new habitats. Experimental tests of the effects of partner-switching on host-symbiont phenotype in this system are required to disentangle the contributions of host genotype, symbiont genotype and their interaction to host-symbiont fitness and local adaptation.

We observed similar responses among hosts in their regulation of symbiont load across light gradients. Consistent with our previous data [28] and a mathematical model of this system [36], we observed that symbiont load per host peaked at low light levels. This occurs because hosts adjust symbiont number to maximise the benefit-to-cost ratio of symbiosis [36]. In the dark, hosts reduce their symbiont load as their maintenance is costly and they provide no benefit to host growth through photosynthesis. At very low light intensities, hosts need many symbionts in order to gain a growth benefit, which albeit costly in terms of demand for nitrogen leads to a peak in symbiont load. As light increases, the per symbiont benefit to hosts increases and so hosts need fewer symbionts to provide the same photosynthetic output, allowing hosts to reduce their N costs. Above a given light level, the per-symbiont benefit saturates leading to an asymptotic relationship between symbiont load and light. The response of symbiont load to light was broadly conserved among our host strains, and our empirical estimates of this trait closely matched the theoretical predictions in Dean et al. [36]. Minor variations in the parameters of the fitted curves were observed but were not associated with variation in dependency, with the exception that symbiont load was highest in the fastest growing and most facultative host (186b) and lowest in the host that was slowest growing and least able to survive without its symbionts (HK1). This suggests that while all host strains have the ability to control symbiont load, an overall higher symbiont load favoured faster symbiotic growth, whereas lower symbiont loads may have evolved in more highly dependent associations, presumably to minimise the costs of symbiosis.

## Conclusions

Comparative evolutionary analysis suggests that host-symbiont dependence varies widely between symbiotic lineages across the tree of life [13]. Data from the study presented here show that the degree of host-symbiont dependence also varies within symbiotic partnerships, and asymmetrically for hosts and symbionts. Where symbiosis is based upon exploitation, as here, our data suggest that the evolution of dependence is less likely in the exploited symbiotic partner, in this case, *Chlorella*.

## Additional files

Additional file 1: **Table S1.** Details of the *Paramecium-Chlorella* strains used in this study. (DOCX 12 kb)

Additional file 2: The raw data that was presented and analysed in the manuscript. (XLSX 42 kb)

Additional file 3: Model outputs for the statistical analyses presented in the manuscript. (DOCX 46 kb)

## Abbreviations

BBM: Bold’s Basal Medium
L:D: Light:Dark
PPM: Protozoan Pellet Media
