# Supplementary material for: Variation and asymmetry in host-symbiont dependence in a microbial symbiosis
Source: BMC Evol Biol. 2018 Jul 9;18:108. doi: 10.1186/s12862-018-1227-9 (PMC6038246; doi:10.1186/s12862-018-1227-9)
Supplement: Supplementary file 1 — Table S1. Details of the Paramecium-Chlorella strains used in this study. (DOCX 12 kb) [file 12862_2018_1227_MOESM1_ESM.docx]

**Table S1**

Details of the *Paramecium-Chlorella* strains used in this study.

| Strain | Year | Location | URL |
| --- | --- | --- | --- |
| 186b | 2006 | Inverawe, UK | **https://www.ccap.ac.uk/strain_info.php?Strain_No=1660/18** |
| HA1 | 2010 | Hirosaki, Japan | http://nbrpcms.nig.ac.jp/paramecium/wp-content/themes/paramecium/data/strain_ha1g.pdf |
| HK1 | 1990 | Chigasaki, Japan | http://nbrpcms.nig.ac.jp/paramecium/wp-content/themes/paramecium/data/strain_hk1g.pdf |
| Dd1 | 1995 | Hitachiota, Japan | http://nbrpcms.nig.ac.jp/paramecium/wp-content/themes/paramecium/data/strain_dd1g.pdf |
| CT39 | - | - | http://nbrpcms.nig.ac.jp/paramecium/wp-content/themes/paramecium/data/strain_ct39g.pdf |
