# Supplementary material for: Variation and asymmetry in host-symbiont dependence in a microbial symbiosis
Source: BMC Evol Biol. 2018 Jul 9;18:108. doi: 10.1186/s12862-018-1227-9 (PMC6038246; doi:10.1186/s12862-018-1227-9)
Supplement: Supplementary file 3 — Model outputs for the statistical analyses presented in the manuscript. (DOCX 46 kb) [file 12862_2018_1227_MOESM3_ESM.docx]

**Host symbiotic growth non-linear regression analysis** – CT39 Removed due to high variance in parameter estimates

Call:

Model: slope ~ (rmax * (light.n - pbar)/(k + (light.n - pbar))) | strain

Data: slopedata[symbionts == "g" & strain != "CT39", ]

Coefficients:

rmax

Estimate Std. Error t value Pr(>|t|)

Dd1 0.2684359 0.07340272 3.657029 1.783458e-04

HA1 0.5160393 0.10411673 4.956353 4.278129e-04

HK1 0.1766509 0.04737774 3.728564 2.325513e-06

s186 0.5753377 0.12187399 4.720759 3.452707e-03

pbar

Estimate Std. Error t value Pr(>|t|)

Dd1 3.78329557 1.1286342 3.35210070 0.0004594226

HA1 -0.09186747 1.0640457 -0.08633789 0.9410697836

HK1 1.15014406 0.9003295 1.27747016 0.0474300915

s186 0.05472349 1.0585332 0.05169747 0.9710145146

k

Estimate Std. Error t value Pr(>|t|)

Dd1 17.015452 9.326754 1.824370 0.03782889

HA1 16.700718 8.884221 1.879818 0.12001068

HK1 8.260467 6.234620 1.324935 0.04034145

s186 19.892866 10.365828 1.919081 0.18823500

Residual standard error: 0.06940574 on 86 degrees of freedom

Note that strain ID s186 is 186b


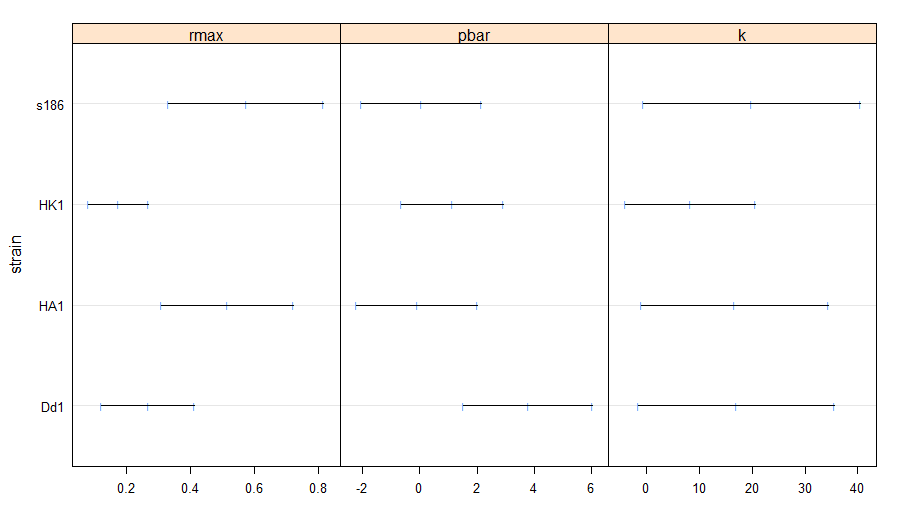


**Host symbiont load non-linear regression analysis**

Call:

Model: host.FL.mn ~ (a * (light.n - l)/(b + ((light.n - l)^c))) | strain

Data: host.FL

Coefficients:

a

Estimate Std. Error t value Pr(>|t|)

CT39 13985847 2584753 5.410903 4.007584e-04

Dd1 21130112 6512990 3.244303 1.051916e-03

HA1 18147382 3985035 4.553883 1.344770e-04

HK1 11308172 2346631 4.818896 4.375117e-06

s186 22705976 3852382 5.894009 1.109982e-06

b

Estimate Std. Error t value Pr(>|t|)

CT39 3.042376 1.1417840 2.664581 0.054343765

Dd1 6.255133 3.5022226 1.786047 0.051360060

HA1 3.554785 1.5239586 2.332599 0.028887430

HK1 2.003719 0.9590379 2.089301 0.017286899

s186 3.425407 1.2591026 2.720514 0.006475345

c

Estimate Std. Error t value Pr(>|t|)

CT39 1.291467 0.05681229 22.73218 4.932328e-15

Dd1 1.590884 0.09991338 15.92263 1.359433e-15

HA1 1.551124 0.07697162 20.15190 1.633347e-16

HK1 1.380785 0.07032434 19.63452 2.668239e-18

s186 1.405294 0.05264168 26.69547 2.754025e-20

l

Estimate Std. Error t value Pr(>|t|)

CT39 -0.2842604 0.1019374 -2.788578 4.479926e-02

Dd1 -0.8925523 0.2754005 -3.240925 1.062175e-03

HA1 -0.4196455 0.1215738 -3.451776 2.138142e-03

HK1 -0.2265049 0.0919472 -2.463423 5.983514e-03

s186 -0.7962617 0.1770086 -4.498435 4.952296e-05

Residual standard error: 588806.4 on 120 degrees of freedom

Note that strain ID s186 is 186b


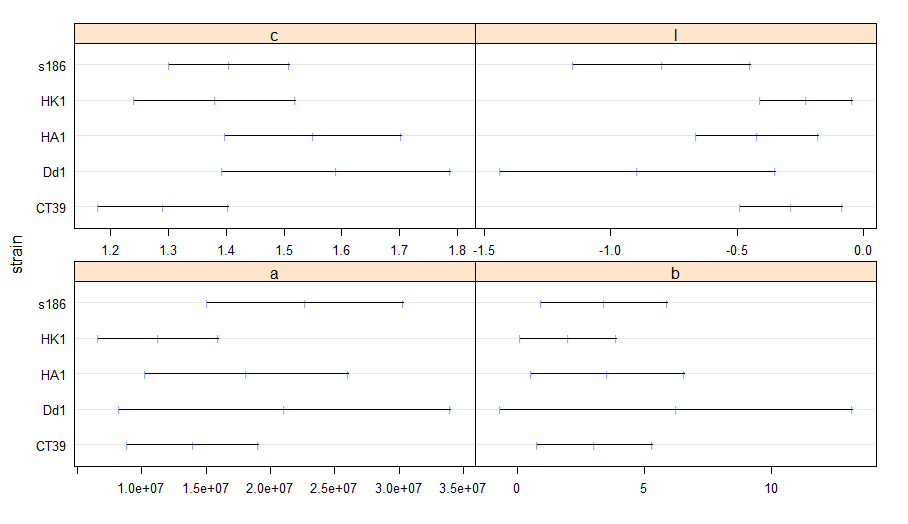


**Host symbiont load non-linear mixed effects model**

Nonlinear mixed-effects model fit by maximum likelihood

Model: host.FL.mn ~ symresp(a, b, c, l, L = light.n)

Data: host.FL

AIC BIC logLik

-373.306 -308.5899 208.653

Random effects:

Formula: a ~ 1 | strain

a.(Intercept) Residual

StdDev: 1.437245e-06 0.05451294

Fixed effects: list(a + b + c + l ~ 1 + strain)

Value Std.Error DF t-value p-value

a.(Intercept) 1.391270 0.256402 116 5.426120 0.0000

a.strainDd1 0.716827 0.697755 116 1.027335 0.3064

a.strainHA1 0.423445 0.473858 116 0.893611 0.3734

a.strainHK1 -0.260401 0.347587 116 -0.749170 0.4553

a.strains186 0.878914 0.462665 116 1.899677 0.0600

b.(Intercept) 3.006673 1.128940 116 2.663271 0.0088

b.strainDd1 3.220454 3.663333 116 0.879105 0.3812

b.strainHA1 0.548015 1.896533 116 0.288956 0.7731

b.strainHK1 -1.002718 1.481365 116 -0.676888 0.4998

b.strains186 0.417267 1.690693 116 0.246802 0.8055

c.(Intercept) 1.290003 0.056674 116 22.761845 0.0000

c.strainDd1 0.300188 0.114771 116 2.615529 0.0101

c.strainHA1 0.261117 0.095585 116 2.731780 0.0073

c.strainHK1 0.090795 0.090320 116 1.005263 0.3169

c.strains186 0.115240 0.077347 116 1.489913 0.1390

l.(Intercept) -0.281630 0.101229 116 -2.782098 0.0063

l.strainDd1 -0.608863 0.292904 116 -2.078713 0.0398

l.strainHA1 -0.138009 0.158200 116 -0.872369 0.3848

l.strainHK1 0.055104 0.136758 116 0.402933 0.6877

l.strains186 -0.514424 0.203878 116 -2.523190 0.0130

Standardized Within-Group Residuals:

Min Q1 Med Q3 Max

-2.94168498 -0.63533970 -0.01036713 0.48430929 2.95636643

Number of Observations: 140

Number of Groups: 5
